# A note on the organization and expression of β-defensin genes in Polish goats

**DOI:** 10.1007/s13353-012-0124-y

**Published:** 2012-11-22

**Authors:** E. Bagnicka, B. Prusak, E. Kościuczuk, J. Jarczak, J. Kaba, N. Strzałkowska, A. Jóźwik, M. Czopowicz, J. Krzyżewski, L. Zwierzchowski

**Affiliations:** 1 Institute of Genetics and Animal Breeding, Polish Academy of Sciences, Jastrzębiec, Postępu 1, 05-552 Magdalenka, Poland; 2 Division of Infectious Diseases and Epidemiology, Department of Large Animal Diseases with the Clinic, Faculty of Veterinary Medicine, Warsaw University of Life Sciences, Nowoursynowska 159c, Warsaw, Poland

**Keywords:** Goat, β-Defensins, Exons, Intron, Sequencing

## Abstract

Nucleotide (cds) and amino acids sequences of the caprine β2-defensin genes were in silico compared to search for the sequence variation and for the *LAP* gene sequences in the goat genome and for the presence of *LAP* gene transcripts in goat tissues. The comparison of the exon sequences revealed that the first 64 amino acids are identical in both LAP and β1-defensin. However, the GBD-1 prepropeptide is shorter by 18 amino acids due to the presence of the stop codon UAA at position 209–211 in *GBD-1* mRNA. The *LAP* gene, which was found, so far, only in Indian goat breeds, is absent in the genome of Polish dairy goats. The introns of the caprine β1- and β2-defensin genes were, for the first time, sequenced; their sequences showed 99.6 % identity, differing in six nucleotide positions.

Three defensins, β1 (GBD-1) and β2 (GBD-2) and lingual antimicrobial peptide (LAP), have been found in goats (*Capra hircus*) until now (Zhao et al. 1999; Sharma et al. 2010). The information on goat *LAP* gene is limited to the mRNA and deduced a.a. sequence (Sharma et al. 2010; GenBank DQ836129; ABG88198.1) found in an Indian goat breed.

The aim of this study was to sequence the introns of caprine β1- and β2-defensin genes and to compare in silico the coding region (cds) and a.a. sequence of the three β-defensins. Moreover, we searched for the *LAP* gene sequence in the genome of Polish dairy goats and the presence of *LAP* gene mRNA in the goat tissues.

The study was carried out on 14 unrelated Polish dairy goats (Polish White Improved and Polish Fawn Improved). The tongue tissues were taken from four 4-month-old male kids immediately after slaughter. The genomic DNA from blood was extracted according to Kanai et al. (1994). To amplify the β-defensin intron sequences, primers were designed based on the goat *GBD-2* cDNA sequence: forward 5′-TCTTCCTGGTCCTGTCTGCT-3′, reverse 5′-CTGTCTAAGGGCGCAGTTTC-3′ (GenBank AJ009877.1). Polymerase chain reaction (PCR) was conducted in the following reaction mixture (50 μl): 50–100 ng of genomic DNA, 200 μM of each dNTP, 1 × PCR buffer, 1.5 mM MgCl_2,_ 0.5 μM of each primer, 1.5 U DNA Taq Gold polymerase (Applied Biosystems). The temperature cycles (40) were as follows: denaturation at 94 °C for 60 s annealing at 68 °C for 60 s, elongation at 73 °C for 40 s. The purified PCR products (about 1,600 bp) were sequenced with the BigDye® Terminator v1.1 Cycle Sequencing Ready Reaction Kit and analyzed using an ABI 3730 DNA Analyzer (Applied Biosystems).

To search for the *LAP* gene sequences in goat genomic DNA and in β-defensin transcripts, PCR was conducted using primers based on the exon 2 of the sequences of the caprine *LAP* and *GBD-1* genes (GenBank DQ836129 and Y17679.1, respectively): forward 5′-AGTCGTCGAAGCTGCCATAG-3′ and reverse 5′-TGTCTAAGGGCGCAGTTTCT-3′. A 174-bp fragment was amplified encompassing the stop codon OCHRE (UAA) in *GBD-1* mRNA or CAA triplet in the *LAP* mRNA (nucleotides 209–211). RT-PCR assay, with the same primers and amplification conditions, was used to explore the presence of the *LAP* gene transcript in tongue tissues of kids. The cDNA and deduced a.a. sequences of goat defensins and cattle *LAP* were aligned with the use of the Mega 4 software (Tamura et al. 2007).

Sequencing of the intron of β-defensin genes revealed six nucleotide mismatches: 575C/T, 576T/C, 950A/G, 1,350G/A, 1,435G/A, and 1,440G/A (positions in sequence obtained in this study; deposited in the GenBank database, accession no. GU119911; Bagnicka et al. 2009). The analysis of nucleotide sequences confirmed that the primers amplified more than one β-defensin gene and we could not state whether the differences in the nucleotide sequences were between β-defensin genes or between animals. The intron sequence of goat β-defensin genes obtained in our study had a similar length (1,478 bp) as that reported for sheep by Luenser et al. (2005) (1,506 bp), but was much shorter than that obtained later by Liu and Jiang (2010) for Chinese goats (1,927 bp).

In the present study, the coding sequences (exons) of goat *GBD-1*, *GBD-2*, *LAP*, and cattle *LAP* genes were in silico compared; also compared were the deduced a.a. sequences of β-defensins 1, 2, and LAP prepropeptides (Fig. 1). The goat *GBD-1* and *LAP* cds and their respective amino acid sequences revealed high similarity. Comparison of cds of goat *LAP* (DQ836129) and *GBD-1* (Y17679.1) genes showed only two variable positions (Fig. 1). We found that the sequence of the first 64 a.a. is identical in goat LAP and GBD-1, but the deduced goat GBD-1 peptide is shorter by 18 amino acids, due to the presence of the stop codon UAA at position 209–211 in GBD-1 mRNA. In the *LAP* mRNA counterpart, this triplet (CAA) encodes glutamine. The length of cattle LAP (NP_982259.3) prepropeptide is the same as for GBD-1, but with 17 differences in the amino acid residues.

**Fig. 1:**
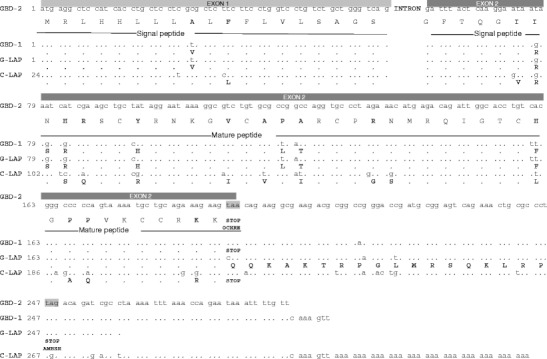
Alignment of the nucleotide sequences of the genes (exons) encoding goat defensins β1 (*GBD-1*), β2 (*GBD-2*), goat LAP (*G-LAP*), and cattle LAP (*C-LAP*), and amino acid sequences of the respective prepropeptides. Differences in amino acids residues are shown in *bold*

Analysis of the gene-specific 174-bp fragments revealed that, in all the investigated individuals, only the *GBD-1* genomic sequences (does) and transcripts (kids) were found, with the stop codon UAA at position 209–211 (Fig. 1). Therefore, our results showed that the *LAP* gene might be absent in Polish dairy goats. The possible explanations for this could be the distinct origin of domestic European and Indian goat breeds. The European goat breeds are mostly descendants of bezoar goats (*Capra aegagrus*), while the Indian breeds come mostly from markhor goats (Devendra 2007). This view was supported by the results of Naderi et al. (2007), who found that mitochondrial DNA haplogroup B was widespread over the whole of Asia, whereas it was very rare in Africa and Europe. This study, for the first time, showed a difference between goat breeds in defensin genes. Moreover, our results suggest that, during evolution, *LAP* and *GBD* genes might have been created by the single mutation from a common ancestor gene, but further studies are needed.
